# The Relationship Between Occupational Stress and Turnover Intention Among Emergency Physicians: A Mediation Analysis

**DOI:** 10.3389/fpubh.2022.901251

**Published:** 2022-06-16

**Authors:** Nan Jiang, Hongling Zhang, Zhen Tan, Yanhong Gong, Mengge Tian, Yafei Wu, Jiali Zhang, Jing Wang, Zhenyuan Chen, Jianxiong Wu, Chuanzhu Lv, Xuan Zhou, Fengjie Yang, Xiaoxv Yin

**Affiliations:** ^1^Department of Social Medicine and Health Management, School of Public Health, Tongji Medical College, Huazhong University of Science and Technology, Wuhan, China; ^2^School of Health and Nursing, Wuchang University of Technology, Wuhan, China; ^3^Shenzhen University General Hospital, Shenzhen University, Shenzhen, China; ^4^Department of Emergency Medicine Center, Sichuan Provincial People's Hospital, University of Electronic Science and Technology of China, Chengdu, China; ^5^Research Unit of Island Emergency Medicine, Chinese Academy of Medical Sciences, Hainan Medical University, Haikou, China; ^6^Key Laboratory of Emergency and Trauma of Ministry of Education, Hainan Medical University, Haikou, China; ^7^Department of Anesthesiology, Tongji Hospital, Tongji Medical College, Huazhong University of Science and Technology, Wuhan, China; ^8^Department of Pediatrics, Tongji Hospital, Tongji Medical College, Huazhong University of Science and Technology, Wuhan, China

**Keywords:** emergency physicians, turnover intention, occupational stress, job satisfaction, China

## Abstract

**Background:**

There is evidence that occupational stress is a risk factor for turnover intentions. However, the structural relationship between occupational stress and turnover intention among emergency physicians has rarely been studied. This study aimed to examine the pathways of occupational stress on turnover intention through job satisfaction and depressive symptoms among emergency physicians in China.

**Methods:**

A cross-sectional study was conducted in China from July 2018 to August 2018. Data were collected using a structured questionnaire that included demographic characteristics, occupational stress, job satisfaction, depressive symptoms, and turnover intention. Hierarchical linear regression was performed to explore the related factors of turnover intention. Structural equation modeling was used to examine the pathways from occupational stress to turnover intention.

**Results:**

A total of 10,457 emergency physicians completed the questionnaire. The score of turnover intention was 11.34 (SD = 3.25), and the average item score of turnover intention was 2.84 (SD = 0.81). In structural equation modeling, the occupational stress not only had a direct effect on turnover intention (standardized direct effect = 0.311, bias-corrected 95% confidence interval [0.261, 0.361], *P* < 0.001), but also had an indirect effect through job satisfaction and depressive symptoms (standardized indirect effect = 0.448, bias-corrected 95% confidence interval [0.412, 0.484], *P* < 0.001). However, the effect of depressive symptoms on turnover intention was weak (standardized coefficient [β] = 0.08, *P* < 0.001).

**Conclusions:**

Job satisfaction partially mediated the relationship between occupational stress and turnover intention. However, due to the weak effect of depressive symptoms on turnover intention, the mediating role of depressive symptoms between occupational and turnover intention had little practical value. It is recommended that hospital administrators prioritize increasing job satisfaction of emergency physicians to reduce the impact of occupational stress on their turnover intention.

## Introduction

The shortage of emergency physicians is an ongoing challenge for healthcare systems worldwide (1). High turnover rates, a major cause of emergency physician shortages, not only significantly increase the operating costs of hospitals (2), but also compromise the quality of medical service (3). The turnover intention is an antecedent variable of turnover behavior (4), which refers to the extent to which employees plan to quit their job (5). It is speculated that measuring the turnover intention of emergency physicians would determine their propensity to leave hospitals (6). Moreover, identifying predictors of turnover intention and formulating corresponding interventions will help reduce turnover intention and even turnover rate of emergency physicians.

Occupational stress is defined as the perception of a discrepancy between environmental demands and individual capacities to fulfill these demands (7). Workplace stress has become an important problem for developing countries (8). Emergency department in China is a stressful workplace, regularly overcrowded and chaotic (9). In recent years, the emergency department overcrowding has become more severe as the patient visits have risen from 51.9 million in 2007 to 166.5 million in 2017 (10). The challenge of providing medical care to such a large volume of patients has added to the already stressful occupational environment (9, 11). Emergency physicians are responsible for making quick decisions and taking crucial actions for critically ill patients in a chaotic environment. Therefore, emergency physicians often face high levels of occupational stress (12). It has been reported that occupational stress is a major organizational factor related to turnover intention (13, 14). A study in Guangdong province of China showed that occupational stress had a direct and positive relationship with physicians' turnover intention (15). In Taiwan, rural physicians who perceived high levels of occupational stress had a stronger intention to leave (16). Accordingly, this study hypothesized that occupational stress could directly affect turnover intention.

Job satisfaction refers to a pleasurable or positive emotional state resulting from the appraisal of one's job (17) and is a significant predictor of turnover intention (18). A German study showed that high job satisfaction contributed to the reduction of physicians' turnover intention (19). A study conducted in China also found that general practitioners with low job satisfaction were associated with higher turnover intention (20). In addition, numerous studies indicated that occupational stress was closely correlated with the job satisfaction of physicians (21–23). We hypothesized that occupational stress could indirectly affect turnover intention through the mediating role of job satisfaction.

Depressive symptoms are a common mental health problem in the global physician population, with a prevalence of 28.8% (24). The prevalence is even higher in China, at 42.3% (25). Chronic unrelieved stress is a key factor in the development of depressive symptoms (26), and higher occupational stress can increase the risk of depressive symptoms (27, 28). Empirical studies have shown that work-related depressive symptoms among physicians were also significantly associated with turnover intention (29). On this basis, this study hypothesized that occupational stress could indirectly affect turnover intention through the mediating role of depressive symptoms.

**Figure 1:**
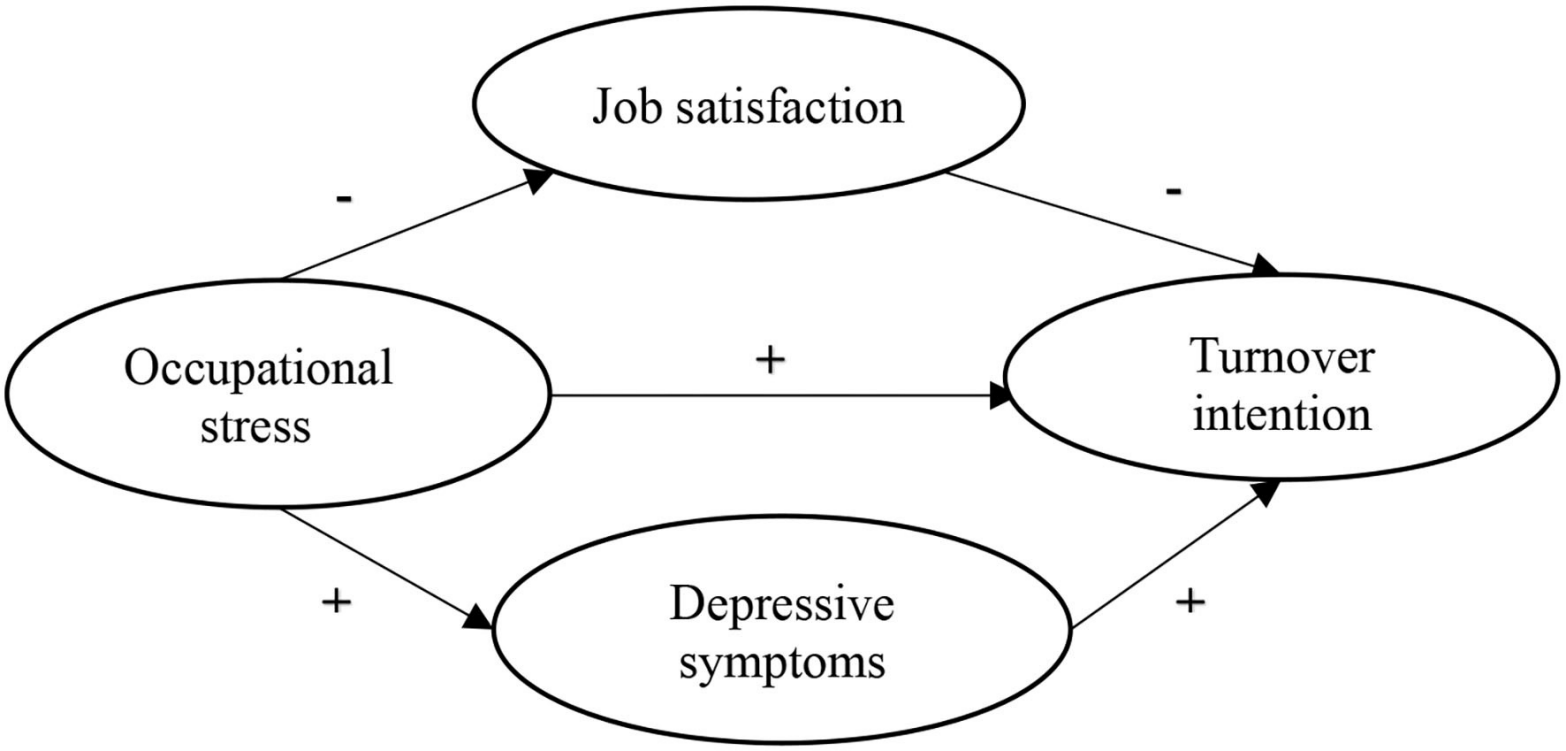
The hypothesized structural model of turnover intention in Chinese emergency physicians.

With increased occupational stress among emergency physicians, it is urgently necessary to identify the pathways by which occupational stress affects turnover intention. Previous studies have mainly explored the pathways from occupational stress to turnover intention in hospital physicians (13, 30), nurses (31), and healthcare workers (32). Few studies have been conducted among emergency physicians. Furthermore, studies conducted on physicians have only explored the association among some of the variables in occupational stress, job satisfaction, depressive symptoms, and turnover intention (13, 30). There is a lack of studies that integrate the structural relationships among them into a comprehensive model. Therefore, this study aimed to explore the mediating role of job satisfaction and depressive symptoms between occupational stress and turnover intention among emergency physicians. Based on the above literature review, we constructed a hypothetical model, as shown in Figure 1.

## Materials and Methods

### Study Design and Participants

This study was part of a national Emergency Survey facilitated by the Medical Administration Bureau of the National Health Commission of the People's Republic of China. Quantitative survey data were collected using the Questionnaire Star (https://www.wjx.cn), an online survey platform. Survey links were sent to the emergency physicians' working platform from July 2018 to August 2018 and were re-sent to the work platform every 7 days to remind until the survey ended. Physicians were invited to complete the electronic questionnaire. Each participant was required to read and agree to the informed consent form before answering the questionnaire. To prevent duplicate submissions, each account is allowed to answer the questionnaire only once.

Inclusion criteria included physicians who worked in the emergency department and volunteered to participate in this survey. Exclusion criteria included interns who had not yet obtained their practicing certificates. During the survey period, a total of 15,288 physicians clicked on the survey link and 10,457 physicians completed the online questionnaire, for a response rate of 68.4%.

### Occupational Stress

The occupational stress was measured by three subscales of the effort-reward imbalance questionnaire: effort, reward and over-commitment (21). Efforts consisted of six items, and each item was rated on a 5-point Likert scale ranging from 1 (strongly disagree) to 5 (strongly agree). The higher the score, the more perceived demands were experienced as stressful. Reward consisted of 11 items and three dimensions: self-esteem, job promotion, and job security. Each item was rated using a 5-point Likert scale that ranged from 1 (strongly agree) to 5 (strongly disagree). The higher the score, the higher the perceived reward. Effort-reward ratio (ERR) was computed according to the formula (21):


(1) $$ERR=\frac{sum\;score\;ofeffort}{sum\;score\;ofreward\times \frac{number\;of\;items\;on\;effort}{number\;ofitems\;on\;reward}}$$

ERR values beyond 1.0 indicated a high amount of effort spent that the rewards received or expected in turn were not met. Over-commitment was measured using a 4-point Likert scale that ranged from 1 (full disagreement) to 4 (full agreement). The higher the score, the more likely a subject was to experience over-commitment at work. Previous studies in Chinese physicians have demonstrated satisfactory reliability and validity of this scale (30, 33). In this study, Cronbach's α for the effort, reward and over-commitment sub-scales were 0.86, 0.93, and 0.86, respectively.

### Job Satisfaction

Job satisfaction was assessed by the Job Satisfaction scale derived from the Leiden Quality of Work Questionnaire (LQWQ) (34). This scale consisted of 6 items and each item was assessed on a 4-point Likert scale, ranging from 1 (strongly disagree) to 4 (strongly agree). A higher score indicated a higher level of job satisfaction. The scale has been proven to have good reliability among emergency physicians (35). In this study, Cronbach's α was 0.86.

### Depressive Symptom

Depressive symptoms were measured using the Patient Health Questionnaire (PHQ-9), which contained nine items. Each item was divided into a four-point degree, with scores ranging from 0 (not at all) to 3 (nearly every day). The total score ranged from 0 to 27. Higher scores indicated the more severe depressive symptoms. The Chinese version of the PHQ-9 scale has been widely applied among Chinese populations and has demonstrated good reliability and validity (36, 37). In this study, Cronbach's α was 0.92.

### Turnover Intention

Turnover intention was measured using the Turnover Intention Scale developed by Kim et al. (38). The scale comprised four items. Each item was assessed on a 5-point Likert scale, ranging from 1 (strongly disagree) to 5 (strongly agree). Reverse scoring was used for the negative items. A higher score indicated a higher likelihood of quitting the current job. The scale has shown good reliability in Chinese healthcare workers (39). In this study, Cronbach's α was 0.84.

### Statistical Analysis

Data analyses were conducted using the Statistical Analysis System (SAS) version 9.4 and Analysis of Moment Structures (AMOS) version 26.0. Descriptive statistics were performed to describe the demographic characteristics and turnover intention of emergency physicians. *T*-tests or one-way analysis of variances (ANOVAs) were conducted to examine the differences in turnover intention across demographic characteristics. Person correlation was used to examine correlations between occupational stress, job satisfaction, depressive symptoms, and turnover intention. Hierarchical multiple regression analysis was performed to explore the association among independent variables with turnover intention. The independent variables were entered in three steps. The characteristics of age, sex, marital status, and educational level were added to the regression model in the first step, occupational stress including ERR and over-commitment was added in the second step. Job satisfaction and depressive symptoms were added in the third step. All differences were assessed using two-tailed tests, and the significance level was set at *P* < 0.05.

Structural equation modeling (SEM) was conducted to assess the mediating effect of job satisfaction and depressive symptoms between occupational stress and turnover intention. The following fit indices were used to assess the overall model fit: the root mean square error of approximation (RMSEA), goodness-of-fit index (GFI), Tucker-Lewis index (TLI), incremental fit index (IFI), and comparative fit index (CFI). GFI, TLI, IFI, and CFI values above 0.90 and RMSEA value below 0.08 indicated acceptable fit (40). The mediating effect was examined using a bias-corrected bootstrap 95% confidence interval (CI). The estimates were based on 5,000 bootstrap samples.

## Results

The characteristics of emergency physicians are shown in Table 1. A total of 10,457 emergency physicians participated in this survey. Of them, 72.98% were male. Nearly one-third of participants aged ≤ 31 years. Most of them were married and attended a bachelor's degree. The proportion of ERR > 1 was 78.39%. In the univariable analysis, there were significant differences in the turnover intention scores in terms of age, sex, marital status, educational level, and ERR (*P* < 0.05).

**Table 1:** Demographic characteristics of the participants (*N* = 10457).

**Characteristics**	**N(%)**	**Turnover intention** **(mean±SD)**	**t/F**	***P***
**Age**				
≤ 31	3111 (29.75)	11.35 ± 3.28	21.92	<0.0001
32~35	2150 (20.56)	11.67 ± 3.27		
36~40	2584 (24.71)	11.46 ± 3.33		
≥41	2612 (24.98)	10.94 ± 3.07		
**Sex**				
Male	7632 (72.98)	11.47 ± 3.32	6.84	<0.0001
Female	2825 (27.02)	11.00 ± 3.04		
**Marital status**				
Married	8828 (84.42)	11.31 ± 3.22	2.07	0.0382
Unmarried/divorced /separated/widowed	1629 (15.58)	11.50 ± 3.41		
**Educational level**				
Associate degree^a^ or lower	1684 (16.10)	10.64 ± 3.17	47.13	<0.0001
Bachelor's degree	7789 (74.49)	11.48 ± 3.24		
Master's degree or higher	984 (9.41)	11.43 ± 3.33		
**ERR**				
>1	8197 (78.39)	11.92 ± 3.21	−42.79	<0.0001
≤ 1	2260 (21.61)	9.25 ± 2.44		

*ERR, effort reward ratio; ^a^An associate degree requires 3 years of education in college after graduation from senior middle school (grade year 10 to 12), or 5 years of education in college after graduation from junior middle school (grade year 7 to 9)*.

Table 2 presents the descriptive data for study variables. The score of turnover intention was 11.34 (SD = 3.25), and the average item score of turnover intention was 2.84 (SD = 0.81). The scores of ERR, over-commitment, job satisfaction, and depressive symptoms were 1.55 (SD = 0.79), 18.26 (SD = 2.67), 12.17 (SD = 3.58), and 8.92 (SD = 5.81), respectively. There were significant correlations between all study variables. All dimensions of occupational stress were negatively correlated with job satisfaction but positively correlated with depressive symptoms and turnover intention. Job satisfaction was negatively correlated with turnover intention. Depressive symptoms were positively correlated with turnover intention.

**Table 2:** Means, standard deviations, and correlations among study variables (*n* = 10457).

	**Mean**	**SD**	**1**	**2**	**3**	**4**
ERR	1.55	0.79	1			
Over-commitment	18.26	2.67	0.59***	1		
Job satisfaction	12.17	3.58	−0.50***	−0.41***	1	
Depressive symptoms	8.92	5.81	0.52***	0.47***	−0.54***	1
Turnover intention	11.34	3.25	0.54***	0.34***	−0.66***	0.50***

ERR, effort reward ratio; ^***^P < 0.001.

The results of hierarchical linear regression are shown in Table 3. The demographic characteristics included in the first step accounted for 1.67% of the variance in turnover intention. In the second step, all dimensions of occupational stress, including ERR and overcommitment, accounted for 16.18% of the variance in turnover intention. In the third step, job satisfaction and depressive symptoms accounted for 28.69% of the variance in turnover intention. When the effects of job satisfaction and depressive symptoms were controlled for, occupational stress on turnover intention was weakened but still significant. It was suggested that job satisfaction and depressive symptoms played mediating roles in the relationship between occupational stress and turnover intention.

**Table 3:** The hierarchical linear regression analysis for turnover intention.

**Variables**	**Turnover intention**		
	**Step1(β^#^)**	**Step2(β^#^)**	**Step3(β^#^)**
**Age (ref:** **≤31)**			
32~35	0.322***	0.101	−0.006
36~40	0.143	−0.113	−0.196**
≥41	−0.232*	−0.462***	−0.176**
**Sex (ref: male)**			
Female	−0.488***	−0.205**	−0.064
**Marital status (ref:) unmarried/divorced/separated/widowed**
Married	−0.237*	−0.382***	−0.322***
**Education level (ref: associate degree or lower)**			
Bachelor's degree	0.740***	0.481***	−0.019
Master's degree or higher	0.693***	0.610***	0.079
**Occupational stress**			
ERR (ref: ≤ 1)			
>1		1.901***	0.422***
Over–commitment		0.303***	0.033**
**Job satisfaction**			−0.476***
**Depressive symptom**			0.103***
**F**	26.4***	253.4***	828.6***
**adjusted R** ^**2**^	0.0167	0.1785	0.4654
**Δ*R*^2^**		0.1618	0.2869

^**#**^Standardized coefficient; *P < 0.05, **P < 0.01, ***P < 0.001 (two-tailed).

**Figure 2:**
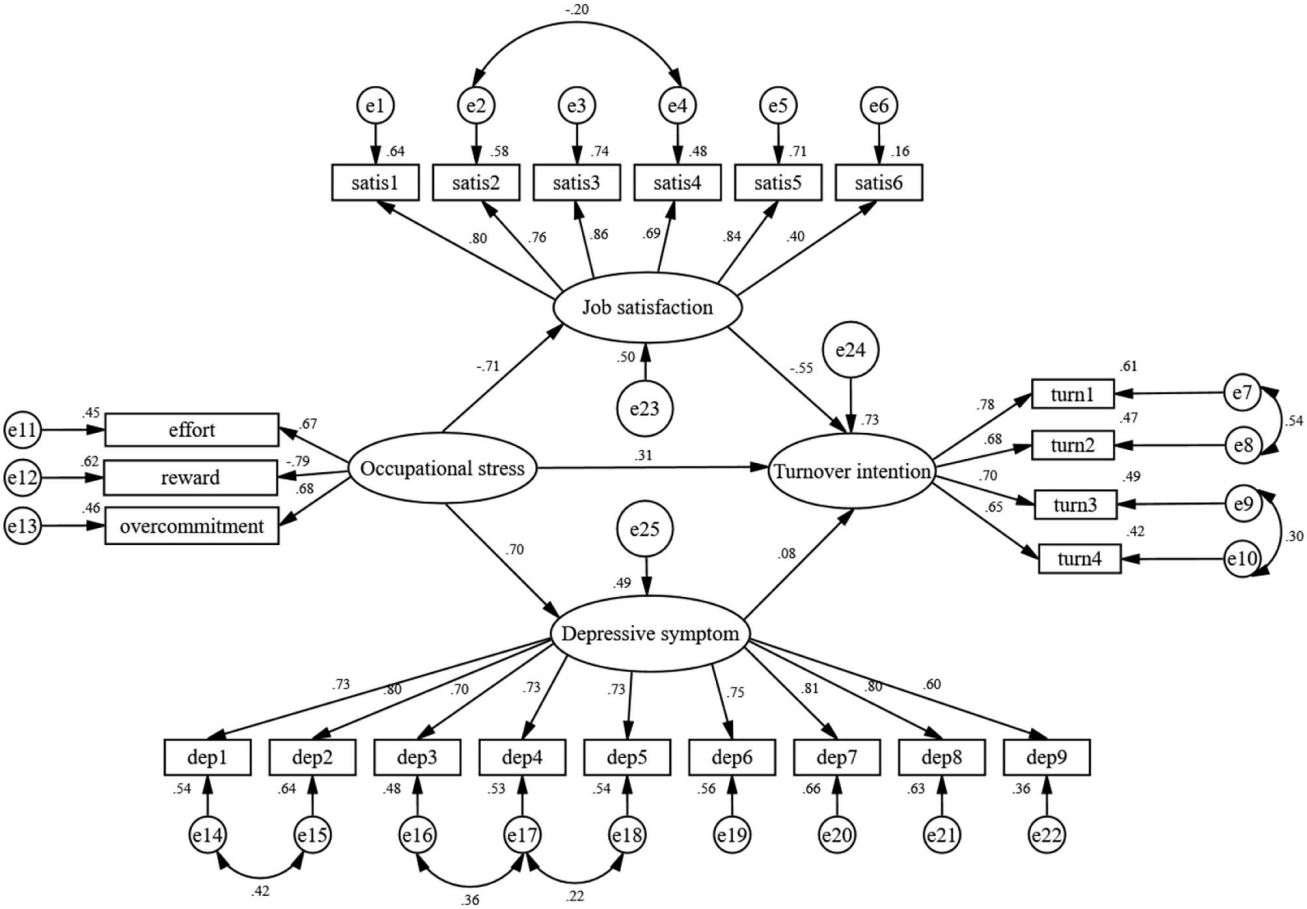
The structural equation model on the relationships between occupational stress, job satisfaction, depressive symptom and turnover intention. Three subscales of the Effort Reward Imbalance Questionnaire labeled effort, reward, and overcommitment. Questionnaire items of the Leiden Quality of Work Questionnaire labeled satis1–satis6. Questionnaire items of the PHQ-9 labeled dep1–dep9. Questionnaire items of Turnover Intention scale labeled turn1–turn4. e1–e22, the measurement error of each observed variable to estimate latent variable. e23–e25, the residual that may affect the endogenous latent variables except the exogenous latent variables.

The structural equation model was constructed to examine the mediating effect of job satisfaction and depressive symptoms. Figure 2 shows the final model with standardized path coefficients. The model-fitting results show that GFI = 0.903, TLI = 0.916, IFI = 0.928, CFI = 0.928 and RMSEA = 0.070. All the above fitting indices were within the acceptable range, indicating that the constructed model fits well. In the SEM, occupational stress was negatively associated with job satisfaction (standardized coefficient [β] = −0.71, *P* < 0.001) and positively associated with depressive symptoms (standardized coefficient [β] = 0.70, *P* < 0.001) and turnover intention (standardized coefficient [β] = 0.31, *P* < 0.001). Job satisfaction was negatively associated with turnover intention (standardized coefficient [β] = −0.55, *P* < 0.001). In addition, depressive symptoms were positively associated with turnover intention (standardized coefficient [β] = 0.08, *P* < 0.001).

The mediating effects were tested by the Bias-Corrected Bootstrap method, and the results show in Table 4. Job satisfaction and depressive symptoms partially mediated the relationship between occupational stress and turnover intention (standardized indirect effect = 0.448, 95% bias-corrected CI [0.412, 0.484], *P* < 0.001).

**Table 4:** Mediating role of job satisfaction on the associations between occupational stress and turnover intention.

**Variables**	Estimates^*^	**Bootstrap**	
		**Bias-corrected 95%CI**	
		**Lower bounds**	**Upper bounds**
Occupational stress → turnover intention (total effects)	0.759	0.738	0.779
Occupational stress → turnover intention (indirect effects)	0.448	0.412	0.484
Occupational stress → turnover intention (direct effects)	0.311	0.261	0.361

* *Standardized estimates*.

## Discussion

This study explored the structural relationships linking occupational stress to turnover intention among Chinese emergency physicians based on nationwide data. The results showed that the average item score of emergency physicians' turnover intention was 2.84, which was higher than that among US physicians (2.67) and Australian healthcare workers (2.06) measured by the same instrument as the present study (41, 42). This difference may be due to variations in the working environment between countries and suggests that hospital administrators should pay more attention to the high turnover intention of Chinese emergency physicians. The key finding of this study was that occupational stress, in addition to directly affecting turnover intention, also indirectly affected turnover intention through job satisfaction. Due to the weak effect of depressive symptoms on turnover intention, the mediating role of depressive symptoms between occupational stress and turnover intention remains to be further validated.

This study found that occupational stress among emergency physicians could directly and positively affect turnover intention. According to a study in Taiwan, occupational stress also had a positive impact on the turnover intention of emergency physicians (43). One possible reason is that emergency physicians often attempt to regulate themselves cognitively or behaviorally when exposed to high-pressure work conditions for a long time (44). The generation of turnover intention or turnover behavior is a way to maintain the stress balance (45). Therefore, hospital administrators should develop appropriate stress reduction programs to promptly correct the effort-reward imbalance of emergency physicians to reduce their turnover intention.

Occupational stress could indirectly influence turnover intention by affecting job satisfaction, which is consistent with the findings of related surveys conducted among dentists and general practitioners (15, 46). This result may be attributable to two aspects. On the one hand, emergency physicians, as the first line of defense of the healthcare system, have to take on heavy workloads and endure poor work conditions (47). It is hard to balance income and benefits with their efforts (9). The long-term effort-reward imbalance could induce adverse emotional reactions in emergency physicians and significantly reduce their job satisfaction (23). On the other hand, emergency physicians are more likely to be overcommitted due to the large workload, which may affect their job satisfaction (24, 48). According to Price's turnover model, structural factors such as occupational stress influence turnover intention through the mediating role of job satisfaction (49). Therefore, improving job satisfaction may be an effective way to reduce turnover intention and retain the emergency physician workforce.

This study also found that occupational stress positively associated with depressive symptoms. Higher occupational stress causes emergency physicians heightened mental concentration, making them more likely to develop mental disorders such as depressive symptoms (50). Previous studies have demonstrated that depressive symptoms were moderately and positively associated with turnover intention (31, 51). However, the effect of depressive symptoms on turnover intention in this study was weak, so the mediating role of depressive symptoms between occupational stress and turnover intention provided little practical value. Further studies are needed to validate this conclusion among emergency physicians to develop effective interventions.

## Limitations

This study also has some limitations. First, this study was based on a cross-sectional survey, and causal relationships between occupational stress, job satisfaction, depressive symptoms, and turnover intention must be interpreted with caution. Second, the results of this study were limited to the group of emergency physicians, and further validation of the structural relationships is needed when extending to other populations. In addition, this study only explored the mediating effects of job satisfaction and depressive symptoms between occupational stress and turnover intention. We did not examine other potential mediating factors. A multi-path structural equation model between occupational stress and turnover intention needs to be constructed in future studies.

## Conclusion

The present study constructed a model to examine the structural relationships between occupational stress, job satisfaction, depressive symptoms, and turnover intention. The results showed that occupational stress not only had a direct effect on turnover intention, but also had an indirect effect, which was partially mediated by job satisfaction. Due to the weak effect of depressive symptoms on turnover intention, the mediating role of depressive symptoms provided little practice value. It is necessary to examine the conclusion in longitudinal studies further. Therefore, to reduce the turnover intention of emergency physicians, hospital administrators should prioritize taking measures to improve their job satisfaction.

## Data Availability Statement

The datasets generated during and/or analyzed during the current study are available from the corresponding author upon reasonable request.

## Ethics Statement

The study was approved by the Medical Ethics Committee of Hainan Medical College (HYLL-2018-035). All participants provided their informed consent before participating in this study.

## Author Contributions

NJ, HZ, FY, and XY performed conception, design, and writing of the manuscript. ZT, YG, MT, YW, and JZ performed data acquisition and literature research. JWa, ZC, JWu, CL, and XZ performed analysis and interpretation of data. All authors contributed to the article and approved the submitted version.

## Funding

This study was supported by the Major Science and Technology Projects (No. ZDKJ202004), Key Research and Development Program (No. ZDYF2020112), Department of Science and Technology of Hainan Province; Natural Science Foundation of Shenzhen University General Hospital (No. SUGH2020QD015), and Shenzhen Natural Science Fund (the Stable Support Plan Program, No. 20200826225552001).

## Conflict of Interest

The authors declare that the research was conducted in the absence of any commercial or financial relationships that could be construed as a potential conflict of interest.

## Publisher's Note

All claims expressed in this article are solely those of the authors and do not necessarily represent those of their affiliated organizations, or those of the publisher, the editors and the reviewers. Any product that may be evaluated in this article, or claim that may be made by its manufacturer, is not guaranteed or endorsed by the publisher.

## Abbreviations

SEM: structural equation modeling
ERR: effort-reward ratio
LQWQ: Leiden Quality of Work Questionnaire
PHQ-9: Patient Health Questionnaire
Amos: Analysis of Moment Structures
SAS: Statistical Analysis System
ANOVA: one-way analysis of variance
CI: confidence interval
RMSEA: root mean square error of approximation
GFI: goodness-of-fit index
TLI: Tucker-Lewis index
IFI: incremental fit index
CFI: comparative fit index
SD: standard deviation.
